# Attitudes and use of medicinal plants during pregnancy among women at health care centers in three regions of Mali, West-Africa

**DOI:** 10.1186/s13002-015-0057-8

**Published:** 2015-10-09

**Authors:** Cecilie Sogn Nergard, Thi Phung Than Ho, Drissa Diallo, Ngolo Ballo, Berit Smestad Paulsen, Hedvig Nordeng

**Affiliations:** Department of Pharmacy, School of Pharmacy, Faculty of Mathematics and Natural Sciences, University of Oslo, PO Box 1065 Blindern, Oslo, N-0316 Norway; Department of Traditional Medicine, National Institute of Research in Public Health, Bamako, Mali; Division of Mental Health, National Institute of Public Health, Oslo, Norway

**Keywords:** Traditional medicine, Medicinal plants, Herbal medicine, Pregnancy, Women’s health, Malaria, Mali

## Abstract

**Background:**

Although, medicinal plants have been important for women’s health historically, the knowledge about such use during pregnancy in developing countries is limited. This is the first quantitative, ethnobotanical study on Malian women’s use of and attitudes towards the use of medicinal plants during pregnancy.

The aim of the study was to describe Malian women’s use of medicinal plants during pregnancy according to indications and to evaluate the potentially safety of such use. The overall aim was to preserve valuable information about medicinal plants for women’s reproductive health for the future.

**Methods:**

Data was collected through structured interviews of 209 pregnant women or mothers in three health care centers in Mali. The women were interviewed about their uses of medicinal plants during pregnancy and their attitudes to such use. Nine specific medicinal plants commonly used in Mali and treatment of eleven common ailments in pregnancy were specifically queried about.

**Results:**

In total, 79.9 % had used medicinal plants during pregnancy. Only 17 women (8.5 %) had received a recommendation from a traditional practitioner (TP). The most commonly used medicinal plants were *Lippia chevalieri* (55.5 %), *Combretum micranthum* (39.7 %), *Parkia biglobosa* (12.0 %) and *Vepris heterophylla* (8.1 %). The most common reasons for use were for well-being (37.7 %), symptoms of malaria (37.1 %) and ”increased salt-elimination” (to reduce edema) (19.2 %). For treatment of symptoms of malaria and urinary tract infections during pregnancy, the women’s choices of medicinal plants agreed with those previously reported from interviews with TPs. Almost 30 % believed that medicinal plants had no adverse effects for the mother.

**Conclusion:**

This study showed an extensive use and knowledge of medicinal plants during pregnancy in three regions in Mali. However, exclusive use of medicinal plants as treatment of malaria and urinary tract infections during pregnancy may pose a health risk for the mother and her unborn child. A wider collaboration with TPs, with local communities and conventional health workers of the health care centers, on the safe use of medicinal plants, is important to promote safer pregnancies and better health care for pregnant women and their unborn infants in Mali.

## Background

Mali is a landlocked country in West Africa with a population of approximately 16 million [1]. Mali has both the second highest birth rate (46 births per 1000 inhabitants) and infant mortality rate (1 death per 9 live births) in the world. Maternal mortality rate is also high with 54 deaths per 10,000 live births. Total fertility rate is 6.25 children born per woman. Physician density is only one per 12,500 inhabitants [1], while there is approximately one traditional medicine practitioner (TP) for every 500 inhabitants [2]. Approximately 75 % of the population is dependent on traditional medicine for primary health care [2]. Medicinal plants are an important part of traditional medicine. The plants are most commonly harvested in the wild and to a smaller extent cultivated by TPs and herbalists. The medicinal plants in the areas investigated, grow on Savanna plains or grasslands between equatorial forests and tropical deserts [3].

Since 1968 medicinal plants in Mali have been studied with one of the primary objectives to establish a mechanism to assure that ethnomedicine is complementary to conventional medicine, assuming that medicines can be produced from local resources, especially from medicinal plants [4]. The Department of Traditional Medicine (DMT) under The National Institute of Research in Public Health, is responsible for this research in Mali, and is a reference center for the West African Health Organization (WAHO) for research on traditional medicine. The WHO has developed a strategy to promote the rational use of traditional medicine with regard to the safety, efficacy, quality and accessibility [2]. The strategy includes building the knowledge base of traditional medicine, to which this ethnobotanical study is intended to contribute. The TPs are regarded to play a key role in the health care system in Mali, and until now the ethnobotanical research on the traditional use of plants for improving healthcare has been based on knowledge held by the TPs. A recently published study shows that TPs in Mali have a broad experience and knowledge about herbal treatment of pregnant women [3]. However, it is not known to what extent women are seeking advice from the TPs on the use of medicinal plants during pregnancy. This is the first study to investigate the knowledge base held by Malian women.

Data on medicinal plants for women’s reproductive health is in general limited and a traditionally ignored subject [5]. The knowledge about the extent of women’s use of herbal medicine during pregnancy is especially scarce in the sub-Saharan Africa [6]. A recent literature review focusing on medicinal plants used for menstrual disorders showed that medicinal plants are widely used in sub-Saharan Africa to treat painful menstruation, to induce or regulate menses, and/or to provoke abortion [7]. The plants used by pregnant women need to be better known in order to ensure that pregnant women receive effective treatment, to identify potentially unsafe use, and also to preserve valuable information about medicinal plants for women’s reproductive health for the future.

The very few studies that have been published on women’s use of medicinal plants during pregnancy from other African countries, such as from Ivory Coast [8], Nigeria [6, 9], Zambia [10], Uganda [11], South-Africa [12], Tanzania [13], Bénin and Gabon [14] indicate a widespread use. However, the botanical data of the majority of these studies as well as information about women’s perceptions and knowledge about these medicinal plants are limited. In the studies from Nigeria and Zambia no medicinal plants were identified. In the western Uganda study almost 500 women, including traditional birth attendants were interviewed and identified 75 plant species for the use during labor. The species were listed, but the frequency of citation of use by informants was not given [11]. In the study from Ivory Coast, 104 women, including eight female TPs, identified 75 plants mainly used to ensure good development of the fetus, facilitate labor, prevent or cure malaria, and prevent miscarriages [8]. This study included the frequency of citation and rank within list*.* In the South-African study, 45 traditional healers were interviewed about the use of plants from a list of established Isihlambezo plants (used to produce traditional herbal decoctions as a preventative health tonic during pregnancy). From this list they cited 10 species as frequently used for treatment of common pregnancy-related ailments, such as edema, indigestion, constipation, infection, high blood pressure and post-partum healing [12]. In the study of 214 pregnant women in Tanzania, a total of 23 medicinal plant species were listed, but no frequency of citations was given [13]. In a study from Bénin and Gabon, that investigated women’s knowledge of medicial plants for reproductive health and childcare, 87 ethnobotanical questionnaires were conducted. The 46 Beninese informants mentioned a total of 248 species for women’s reproductive health, while the 41 Gabon informants mentioned a total of 189 species for women’s health. All species mentioned and their use were recorded [14]. Frequency of citation was only given for the most frequently cited plant species. Typically, the same plant was mentioned used for several types of ailments.

As far as we know, no quantitative study on Malian women’s use of medicinal plants during pregnancy has ever been conducted. This study is conducted from a woman’s perspective with the overall aim to describe the use of medicinal plants among pregnant women in Mali.

In developed countries reasons for preference of herbal medicine has been associated with different socio-demographic characteristics. A multi-national study showed that women using herbal medicines during pregnancy were characteristically having their first child, they were current students or had an education other than high school degree. Most commonly the women self-medicated with herbal medicine to treat pregnancy-related health ailments [15]. In Norway a higher proportion of users of herbal medicine during pregnancy had tertiary education [16], prior use of herbs, high knowledge about herbal medicines and age between 26 and 35 years [17]. In Australia, women who self-prescribed herbal medicine during pregnancy were more likely to live in a rural environment [18]. Mother’s age may serve as a proxy for the women’s accumulated knowledge of health care services, but on the other hand because of development in educational opportunities for women, younger women might have an enhanced knowledge of modern health care services and place more value upon modern medicine [19]. In developing countries it is often assumed that the reasons for herbal use is associated with cultural and personal believes, the hight cost and low accessibility to conventional medicine and health care. Studies focusing on socio-demographic variables have divergent results. A study in Kenya showed that women with no formal or only primary education used herbal medicine more than women with higher eduction [20]. In one study from Nigeria the level of education had no impact on the usage of native herbs [21], however another study showed that women who (or whose spouses) had higher education were less likely to use herbs. Unmarried women and attendees of the rural center were also more likely to use herbal decoctions [22]. Several studies have failed to show any difference in socio-demographic characteristics between users and non-users in developing countries [10, 23]. Based on these previous findings, we hypothesized that the woman's socio‐demographic characteristics might be related to use of medicinal plants during pregnancy. In specific, we aimed to identify and describe the most frequently used medicinal plants in pregnancy, and study indications of use and women’s perception of their effect and safety in pregnancy.

## Methods

This study is a quantitative, ethnobotanical interview-study including a descriptive part, of 209 pregnant women or mothers of infants in three regions in Mali; one urban region (Sub-District of Daoudabougou, population size approximately 27,000, in Bamako – the capital of Mali) and two rural regions (Siby, 50 km. south of Bamako, population size approximately 27,000 and Dioila, commune of Banco, 130 km. east of Bamako, population size approximately 29,000). These regions were selected due to the geographic proximity to the Department of Traditional Medicine at the National Institute of Research in Public Health in Bamako and previous collaboration with the TPs in these regions [24–26].

The women were recruited while visiting the community health care center in their region (CsCom ASACODA in Bamako, ScCom Siby in Siby, Reference center of the Health district of Dioila in Dioila), either for prenatal check-ups or for the infant vaccination program. All women who visited the centers during the visit of the research team were asked to participate in the study. The women were told the purpose of the study prior to inclusion, and 209 out of the 211 invited women, accepted to participate. The interviews were performed during the period October to December 2011, in Bambara, the local language, at the health care centers with an interpreter from The Department of Traditional Medicine at the National Institute of Research in Public Health. The interviews were performed with the goal of preserving and increasing the knowledge of medicinal plants commonly used during pregnancy.

The interview was divided into four parts in the following order:

1. Questions about the woman’s health and description of any chronic disease and medicine or herb used. The question was as follows “Do you suffer from any chronic disease, for instance asthma, allergy or malaria?” We specifically asked about malaria as in a similar study from Benin and Gabon [14], malaria in pregnancy was frequently cited by women as a chronic disease and a health concern which was treated with medicinal plants.
2. The woman’s experience with any of eleven common ailments in pregnancy, namely *nausea, tiredness, heartburn problems, skin problems, common cold, urinary tract infection, other infections, constipation, pain in back, neck or shoulder, headache and sleeping problems* and if she had used any treatment against it. In addition, she was asked whether she had had any other diseases or ailments than the eleven specifically mentioned, and if yes, the name and any treatment received.
3. The use in pregnancy of nine specific commonly used medicinal plants, namely *Opilia amentacea*, *Ximenia americana*, *Cola cordifolia*, *Combretum glutinosum*, *Parkia bigolobosa*, *Trichilia emetica*, *Combretum micranthum*, *Lippia chevalieri* and *Vepris heterophylla,* and who had recommended the use. These nine plants are used extensively by TPs for pregnancy related ailments [3], and were selected based on prior ethnopharmacological studies in Mali [24–26]. The medicinal plants were presented to the women by mentioning the local names of the plants (Appendix 1: Table 4).
4. The women’s perception of risk and efficacy of traditional and conventional medicines for the mother and the unborn child.

The women received a small monetary sum (2000 CFA, approximately 4 US dollars = 3 Euro) and 20 cola nuts as is tradition in the Malian culture as gratitude for participating in the study and for their time spent.

Descriptive statistics with the Pearson Chi-square test were used to compare differences in proportions in the demographic characteristics (age, marital status, area of residency, level of education, occupation and parity) between women using medicinal plants in pregnancy and women not using any medicinal plants in pregnancy. When the numbers in any given cell was below 5, we used the Fisher Exact Test. A p-value of <0.05 was considered statistically significant. Statistical analysis was performed using the Statistical Package for the Social Sciences (SPSS) version 22.0 (IMB SPSS Statistics, Armonk, USA).

Voucher specimen were collected and identified after the interviews, with help from the guide of the locality and by the team of the Department of Traditional Medicine. Samples are deposited at the Department of Traditional Medicine, Bamako, Mali (Appendix 1: Table 4).

The study was conducted in accordance with international, national and institutional rules concerning the biodiversity rights. The Study was approved by the Regional Ethics Committee in Norway (REC Region South-East) and by the Norwegian Social Science Data Services (NSD). All computerized data were handled and stored anonymously.

## Results and discussion

### A widespread use of medicinal plants

A total of 209 women were interviewed during the eight weeks study period in Siby, Dioila and Bamako. When the women were asked whether they had taken any medicinal plant or not at the beginning of the interview, only 31.6 % (66 of 209) confirmed taking at least one herbal medicine during pregnancy. However, after being asked about the use of nine specific commonly used medicinal plants, 79.9 % (167 of 209) replied that they had been using one or several of these medicinal plants. The demographic profile of the users and non-users are given in Table 1. In our study, we found that maternal demographic characteristics were not associated with use of medicinal plants in pregnancy. This might be due to low statistical power to detect true differences in demographic characteristics between users and non-users, or indicate that use of medicinal plants are so widespread in society in Mali, that demographic factors do not impact on use of medicinal plants.

**Table 1:** Demographic characteristics of the study population according to use of medicinal plants in pregnancy, *n* = 209

	Total	Women who used medicinal plants during pregnancy	Women without use of medicinal plants during pregnancy
	*n* = 209	*n* = 167	*n* = 42
	*n* (%)	*n* (%)	*n* (%)
Age (years)			
≤ 20	64 (30.6)	47 (28.1)	17 (40.5)
21-25	64 (30.6)	53 (31.7)	11 (26.2)
26-30	32 (15.3)	26 (15.6)	6 (14.3)
≥ 31	41 (19.6)	36 (21.6)	5 (11.9)
Unknown	8 (3.8)	5 (3.0)	3 (7.2)
Marital status			
Married/Cohabitant	203 (97.1)	161 (96.4)	42 (100.0)
Other	6 (2.9)	6 (3.6)	0
Area of residents			
Rural (Siby, Dioila)	150 (71.8)	46 (27.5)	13 (31.0)
Urban (Bamako)	59 (28.2)	121 (72.5)	29 (69.0)
Level of education^a^			
None or very low	97 (46.4)	75 (44.9)	22 (52.4)
Low	89 (42.6)	70 (41.9)	19 (45.2)
High	23 (11.0)	22 (13.2)	1 (2.4)
Occupation			
Housewife	169 (80.9)	135 (80.8)	34 (81.0)
Other	40 (19.1)	32 (19.2)	8 (19.0)
Parity			
0	52 (24.9)	36 (21.6)	16 (38.1)
1	41 (19.6)	33 (19.8)	8 (19.0)
>1	116 (55.5)	98 (58.7)	18 (42.9)

^a^Level of education refers to the highest level of education obtained;”none or very low” indicates no formal education/school,”low” indicates middle school or less, and”high” refers to high school or university

There were no significant statistical differences in demographic characteristics between women who used medicinal plants in pregnancy compared to women who did not use medicinal plants in pregnancy (Chi-square test, *p* < 0.05 or Fisher Exact Test, *p* < 0.05 when the number < 5 per cell

Results from studies from other developing countries diverge from the percentage of women reporting having used medicinal plants during pregnancy from 37 % in Taiwan [27], 55 % in South-Africa [28], 56 % in Hong Kong [29], 68 % in Nigeria [6], to 93.6 % in another study in South-Africa [12]. It is difficult to ascertain whether differences in prevalence are caused by differences in study design and methodology or whether they represent true differences in herbal medicine use. The cultural heritage, economic situation and access to conventional medicines and health care services may have an important impact. In Mali approximately 75 % of the population is dependent on traditional medicine for primary health care [2]. Although the women in our study did have modern health facilities available to them, still 80 % chose to use herbal medicine for their ailments.

Our findings indicate the importance of naming specific ailments and medicinal plants to capture their use. The personal interview helped making the women aware that plants they may not have regarded as medicinal plants were defined as such. Medicinal plants were also used based on tradition, and in many cases this was without the use being regarded as treatment, for example medicinal plants being used for well-being and against tiredness. Although the use of these medicinal plants may not be regarded as treatment as such, both mother and fetus will be exposed to these plants when used during pregnancy for which effects are largely unknown.

In total, the women reported 369 incidences of medicinal plant use during pregnancy. The women reporting using medicinal plants used on average 2.2 different medicinal plants each (median: two medicinal plants each). The highest number of medicinal plants used was 12, which was reported by two women. An overview of the most commonly used medicinal plants and their indications is shown in Table 2.

**Table 2:** Overview of the most frequently used medicinal plants during pregnancy; their preparations and indications

Medicinal plant (local name): Preparation	No. of women with citations, *n* = 167 (%)	Most common indications (citations) per indication
*Lippia chevalieri* (N’ganibakala): Drink boiled decoction of plant bundles or leaves	116 (69.5)	Well-being (50) Nutrition or as a dietary supplement (39)
*Combretum micranthum* (N’golobè): Drink boiled decoction of plant bundles or leaves	83 (49.7)	Symptoms of malaria (48) Edema (20)
*Parkia biglobosa* (Néré): Eat fruits or drink boiled decoction or cold maceration of stem bark.	25 (15.0)	Well-being (12) Urinary tract infection (6) Symptoms of malaria (3)
*Vepris heterophylla* (Kita kinkeliba): Drink boiled decoction of plant bundles, mainly leaves	17 (10.2)	Symptoms of malaria (6) Constipation (4) Edema (3)
*Stylosanthes erecta* (Sekoufali): Drink boiled decoction of plant bundles, mainly leaves	14 (8.4)	Symptoms of malaria (12) Tiredness (2)
*Ximenia americana* (Ntonkè): Eat fruits or drink boiled decoction of plant bundles, mainly leaves. Vapor may be inhaled	14 (8.4)	Well-being (6) To increase appetite (2)
*Mitragyna inermis* (Djun): Drink boiled decoction of leaves or plant bundles and leaves.	13 (7.8)	Symptoms of malaria (12) Urinary tract infection (1)
*Combretum glutinosum* (Ganianka): Drink boiled decoction of plant bundles, mainly leaves	11 (6.6)	Symptoms of malaria (3) Tiredness (3)
*Opilia amentacea syn. Opilia celtidifolia* (Korôgé): Drink boiled decoction of plant bundles (mainly leaves) or leaves	10 (6.0)	Symptoms of malaria (7) To increase appetite (2)
*Cola cordifolia* (N’tabanoko): Eat fruits or drink boiled decoction of plant bundles (mainly leaves), vapor may be inhaled	8 (4.8)	Well-being (6) Symptoms of malaria (1) To increase appetite (1)
*Trichilia emetica* (Soulafinzan): Drink boiled decoction of plant bundles, mainly leaves	6 (3.6)	Malaria (5) Tiredness (1)

Eight women used medicinal plants throughout the pregnancy, while others reported treatment periods of one to two days (*n* = 23), 3 to 7 days (*n* = 55), 8 to 14 days (*n* = 14) or more than 14 days (*n* = 36). As many as 74 women replied that they used the medicinal plants until they felt better, without specifics about their treatment period. The treatment periods depended on the specific herb used and the condition treated. Different parts of the plants were used depending on the perceived need. The use of bundles (leaves and stem bark together) was most common (60.7 %), followed by leaves only (30.8 %), fruits (8.1 %) and stem bark (5.6 %). The majority of preparations were in form of decoctions (76.6 %).

### The most commonly used plants

Of all the incidents of medicinal plant use, 162 out of 369 (43.9 %) happened during the first trimester; *Lippia chevalieri* (*n* = 67) and *Combretum micranthum* (*n* = 50) were most commonly used. Considering all trimesters together, *Lippia chevalieri* (*n* = 116) and *Combretum micranthum* (*n* = 83) were, by far, the most commonly used medicinal plants (Table 2). In Africa, but also in other parts of the world, the leaves from *Lippia* spp. have traditionally been used to make aromatic teas. In rural areas in West Africa, the tea is typically drunk after a long working day to relax and to improve sleep, while in the urban areas the tea is often taken in the morning to relieve stress for the coming day [30]. Neither the constituents of *L. chevalieri* nor any biological activities, have been thoroughly investigated. It is therefore impossible to make any predictions about possible effects of *L. chevalieri* on a pregnant woman or the fetus. Leaves from *Combretum micranthum* are used in West Africa as a general remedy for the treatment of different kinds of diseases. One of the local names “Kinkeliba” has become a synonym for “medicine” in several languages [24].

### Medicinal plants used against malaria

Symptoms of malaria were frequently cited by the women to be treated with medicinal plants (Table 2). *C. micranthum* was the plant most commonly mentioned (*n* = 48). Several other plants were also reported used for this indication; *Stylosanthes erecta* (*n* = 12), *Mitragyna inermis* (*n* = 12), *Opilia amentacea* (*n* = 7), *Vepris heterophylla* (*n* = 6), *Trichilia emetica* (*n* = 5), *Combretum glutinosum* (*n* = 3) and *Parkia biglobosa* (*n* = 3). The use of these specific plant preparations is highly consistent with findings in a previous study where TPs in Mali were interviewed about which medicinal plants they used to treat symptoms of malaria during pregnancy [3]. Nordeng et al. reported a high consensus for *Combretum micranthum* (leaves), *Trichilia emetica* (leaves or root), *Vepris heterophylla* (leaves), *Lippia chevalieri* (leaves or root) and *Parkia biglobosa* (stem bark, leaf or fruit) against malaria [3]. These plants have also previously been reported used traditionally in Africa against malaria or fever, but research on their potential biological activities is limited. Decoction of roots or leaves of *Combretum micranthum* is widely used in Africa as an antipyretic [24], which may explain its use against symptoms of malaria. However, results from in vitro studies on antimalaria activities are conflicting [31–34], and there are no studies that elucidate any potential effects on a pregnancy. In vitro studies of antimalarial activities of *Mitragyna inermis* and *Opilia amentacea* encourage further investigations [35, 36]. Leaves and roots from *T. emetica* is extensively used in Africa as an antipyretic and as a remedy to alleviate pain [37]. In vitro studies suggest that *T. emetica* has anti-inflammatory and analgesic properties [38], but the results from in vitro studies on potential antimalarial activities are conflicting [37]. Toxicity data are limited, but water extracts of the roots have shown toxicity against the brine shrimp larvae [39]. A kurubasch aldehyde from *T. emetica* inhibited growth of cancer cell lines [40], and one extract exhibited hormonal influences on prostate cancer cells [41]. Leaves of the plant were found to cause death in laboratory guineapigs due to edema of the lungs, while an aqueous extract did not exhibit cytotoxic effects against brine shrimp [30]. The potential cytotoxic, antiproliferative and hormonal effects urge for caution, especially concerning the root of *T. emetica*, and the use by pregnant women should be discouraged.

It is of concern that medicinal plants are used by some pregnant women as the only treatment against malaria. Of the 62 women treating symptoms of malaria with medicinal plants, only eight (12.9 %) had been recommended to do so by a TP and over 80 % based this use on advice from family and friends or used them on their own initiative (82.3 %). No doctor had recommended such use. Africa remains the region with the highest burden of malaria cases and deaths in the world. Malaria during pregnancy often contributes to maternal anemia, premature delivery, and low birth weight, thereby leading to increased child mortality [42]. Ideally, conventional malaria medicines should be used according to the WHO-guidelines [43], but for many women in Mali medicinal plants may be the only alternative. In a focus group discussion in Mali pregnant women without money resorted to herbal medicine for the treatment of malaria [44]. It is therefore also important to gain more scientific data on these plants that are widely used by pregnant women and collaborate with TPs in order to promote safer pregnancies and better health care for pregnant women and their unborn infants.

### Women’s common ailments during pregnancy and treatment

In total, 199 women (95.2 %) reported experiencing one or more pregnancy related ailments, most commonly tiredness (76.0 %), reflux (63.5 %) and fever (61.5 %) (Table 3).

**Table 3:** Frequent ailments in pregnancy according to treatment status

Ailment	Women with ailment	Any treatment	Treatment with medicinal plants
	*n* (% of total)	*n* (% of women with ailment)	*n* (% of women with ailment)
Tiredness	158/208 (76.0)	68/156 (43.6)	5/156 (3.2)
Reflux	132/208 (63.5)	52/120 (43.3)	5/120 (4.2)
Fever	128/208 (61.5)	79/127 (62.2)	1/127 (0.8)
Headache	124/208 (59.6)	88/123 (71.5)	2/123 (1.6)
Common cold	128/209 (61.2)	79/127 (62.2)	1/127 (0.8)
Pain in back, neck, shoulder	124/208 (59.6)	33/123 (26.8)	0 (0.0)
Nausea	112/207 (54.1)	73/112 (65.2)	3/112 (2.7)
Urinary tract infection	98/207 (47.3)	84/98 (84.8)	14/99 (14.0)
Constipation	93/207 (44.9)	20/91 (22.0)	4/91 (4.4)
Sleep problems	26/207 (12.6)	2/25 (8.0)	0 (0.0)
Skin disorder/itching	20/209 (9.6)	6/20 (30.0)	0 (0.0)

Missing information on treatment status: tiredness (*n* = 2), reflux (*n* = 12), fever, (*n* = 1), headache (*n* = 1), common cold (*n* = 1), pain in back, neck, shoulder (*n* = 1), constipation (*n* = 2), sleep problems (*n* = 1)

On average, they reported five ailments each. The conditions were treated in different ways and to a variable extent. Urinary tract infections were the conditions most commonly treated (84.4 % of the cases), followed by headache (71.5 %), nausea (65.2 %), fever (62.2 %), while the remaining conditions were treated in less than 50 % of the cases. The highest proportion of women using medicinal plants for treatment of these common ailments was found for the treatment of urinary tract infection (UTI). Of the 98 women who treated their UTIs, 14 had used medicinal plants as treatment (14.0 %) with *Parkia biglobosa* as the most frequently cited plant (*n* = 6). This is accordance with *P. biglobosa* being the most frequently cited medicinal plant for the treatment of UTI in pregnancy by the TPs in the same area [3]. In a preliminary study stem bark extracts from *P. biglobosa* showed antimicrobial activities in vitro against several bacterial isolates, including *Escherichia coli* [45]. As *E. coli* is the main pathogen involved in UTIs, these results are encouraging for future studies. However, clinical trials proving effect against UTI is currently lacking. Bacteriuria is associated with an increased risk of preterm birth, intrauterine growth restriction and low birth weight [46]. Since these consequences of UTI on pregnancy outcomes are well documented, guidelines on UTI in pregnancy clearly state that UTI should always be treated with antibiotics [47]. If women with access to antibiotics choose to treat UTI with medicinal plants as replacement for antibiotics, this may have severe negative consequences for pregnancy outcome. This practice should strongly be advised against. Moreover, we believe that there is an urgent need of studies on effect and safety of medicinal plants that are commonly used by pregnant women in developing countries, especially those used for potentially dangerous diseases as malaria and UTI.

### Women’s sources of advice for the treatment of ailments during pregnancy

Use of medicinal plants was most commonly initiated after recommendations from friends and family (36.2 %) or on the woman’s own initiative (32.2 %). Many women explained that their main reason for trusting such advice was that they knew the person well. Only 8.5 % of the women had been recommended the use of herbal medicine by a TP. When asked about preferred sources of information about treatments in pregnancy, 48 % (95 of 198 women in need of information) preferred to seek advice from family or friends and 55 (27.8 %) from TPs. In the study from Bénin and Gabon, the women also mostly did self-medication instead of consulting a TP [14]. In our study only one of the women would ask a medical doctor, only three a midwife, while 38 women would never ask for advice even though they had information needs.

### Women’s attitudes

In total, only 8 % of the women preferred herbal medicine to conventional medicine. They found herbal medicine to be more efficient. Almost one third (31.7 %) were of the opinion that both traditional and conventional medicines were equally effective, while 43.1 % found that herbal medicine was less effective than conventional medicine. Twenty-four women did not have any opinion about the use of herbal medicine during pregnancy, and only two women were of the opinion that herbal medicine should not be used. Those women who were positive to the use of herbal medicine explained that medicinal plants are effective in the treatment of diseases and have less adverse effects than conventional drugs. Some of the comments were:

*“Traditional medicines are more important than conventional medicines because they cure diseases more effectively. All pregnant women should use medicinal plants to treat their diseases”*

*“Conventional medicines only have a temporary effect, while traditional medicines cure diseases and are much cheaper.”*

Among those who were negative to the use of medicinal plants, they pinpointed that herbal drugs are not pure or clean; they give more adverse effects and have no clearly defined dosages. Some of the comments were:

*“Conventional medicines are superior because they are effective and efficient.”*

*“I am afraid to use traditional medicine during pregnancy because it lacks defined dosage form as is the case for conventional medicine.”*

*“I don’t like traditional medicine because it tastes bitter and can make you throw up. I prefer conventional medicine because it cures diseases quicker.”*

Several women (*n* = 60) believed that medicinal plants could not cause any adverse effects for the mother. However, 18 women thought that use of traditional medicines could lead to birth complications or induce abortion. A few women (*n* = 6) were concerned that combining traditional and conventional medicine could cause complications and adverse effects. Many women did not have any opinion whether there could be a risk associated with the use of medicinal plants during pregnancy for either the mother (17.7 %) or the unborn child (23.9 %). During the interview almost 50 % of the women expressed that it is important to treat diseases of the mother during pregnancy. Among these women, the knowledge of adverse effects from medicinal plants was limited. Uncritical use of medicinal plants during pregnancy can pose major risks to both the fetus and the mother.

This study shows that these pregnant women used medicinal plants largely unsupervised. This may be due to several factors such as a cultural tradition, the local communities having a broad knowledge about medicinal plants, high costs of conventional medicines, and low access to health care in general. However, in this study even though the women had modern health care available and an information need, about 20 % of them still would not ask for a doctor’s advice. This might be due to a high trust and reliance on TPs or potentially a distrust or lack of communication between a doctor and pregnant women when it comes to use of medicinal plants. The common use of medicinal plantsobserved warrants special attention due to their unknown efficacy and risks. It seems important to establish a wider collaboration between the local communities, the TPs and conventional health workers of the health care centers on the safe use of medicinal plants during pregnancy. Doctors’ communication with the pregnant women regarding medicinal plants may also need to improve. Future research should prioritize the characterization of constituents and the biological activities of the most commonly used medicinal plants among pregnant women. The plants presented in this study should be included in a much needed, comprehensive list or pharmacopeia of medicinal plants used traditionally. In that way, this study will contribute to perpetuating knowledge that is at risk of being lost.

There are some limitations to the study that should be acknowledged. This study was conducted in three regions in Mali with a limited number of participants, and may therefore not be representative of the entire country. The majority of the women interviewed were from the urban part, Bamako. However, we did not find any differences between users and non-users in the socio-demographic variables. Since this is a retrospective study, it may be subjected to recall bias. Cultural beliefs may also have an impact, as Malian women usually don’t talk about their pregnancy and they keep it a secret until they cannot longer hide it. This may have led to an underestimation of their use of medicinal plants. This study has also several strengths. Firstly, the personal interview with its structured questionnaire and specific naming of several different medicinal plants, contributed to making the women aware that plants they did not regard as medicines could be defined as such. Another important strength is that both a botanist and an anthropologist from Mali participated during all the interviews. Their knowledge about the culture, local language and medicinal plants was essential for the interviews with the women and clearly contributed to improving the quality of the data collected. Our results should be interpreted with these advantages and limitations in mind.

## Conclusion

This study showed an extensive use and knowledge of herbal medicine during pregnancy among women visiting health care centers in three regions in Mali. However, the medicinal plants were used largely unsupervised and many women did not recognize that medicinal plants could potentially have adverse effects on her pregnancy. The use of medicinal plants only, as treatment of diseases like malaria and urinary tract infections, may pose a health risk for the mother and her unborn child. A wider collaboration with the local communities, TPs, and health care centers regarding the safe use of medicinal plants, could promote safer pregnancies and better health care for pregnant women and their unborn infants in this African country.

## Appendix 1

**Table 4:** Plant species cited by more than one of the participants, in alphabetic order: Scientific name, local plant name(s), plant part used, preparation, use category and voucher specimen number (DMT #) located in the herbarium of the Department of Traditional Medicine at National Institute of Research in Public Health, Mali

*Plant name (author name)*	Local name(s)	*Part used*	*Preparation*	*Use category*	*DMT #*
*Sarcocephalus latifolius* (Sm.) E.A.Bruce	Baroufou, Badi	*stem bark, roots, leaves*	*DC, powder*	*UTI, malaria-symptoms*	1117
*Vitex doniana* Sweet	Kouronifing	*leaves*	*DC*	*nausea, malaria-symptoms*	*1893*
*Pteleopsis suberosa* Engl. & Deils	Terenifou	*bark*	*DC*	*UTI*	0722
*Eclipta prostrata (*L.) L.	Moussofing	*leaves*	*DC*	*UTI, malaria-symptoms*	*2395*
*Chrysopogon nigritanus* (Benth.) Veldkamf	Babi	*leaves*	*DC*	*increase salt-elimination, malaria-symptoms, nausea*	*3764*
*Cyperus rotondus* L.	Genni	*leaves*	*DC*	*malaria-symptoms*	*1077*
*Butyrospermum parkii* Kotschy	Si tulu	*fruit*	*butter*	*tiredness, UTI*	*1425*
*Cola cordifolia* (Cav.) R.Br.	N’tabanoko/Ntaba	*fruit, bundles (mainly leaves)*	*eating fruit, DC, vapor*	*well-being, symptoms of malaria, increase appetite*	*1331*
*Combretum glutinosum* Perr.	Cangara	*Plant bundles (mainly leaves)*	*DC*	*malaria-symptoms, tiredness*	0533
*Combretum micranthum* G.Don.	N’golobé	*Plant bundles or leaves*	*DC*	*malaria-symptoms, edema*	0587
*Guiera senegalensis* J.F.Gmel.	Kundje	*leaves*	*DC*	*malaria-symptoms*	0749
*Lippia chevalieri* Mold.	N’ganibakala	*leaves or bundles*	*DC*	*well-being, nutrition, dietary supplement*	0001
*Mitragyna inermis* (Willd.) O.Kze.	Djun	*leaves or bundles and leaves*	*DC*	*malaria-symptoms, UTI*	2263
*Opilia amentacea* Roxb. (syn. *Opilia celtidifolia*) (G. et Perr.) Endl.	Korôgé	*bundles (mainly leaves) or leaves*	*DC*	*malaria-symptoms, to increase appetite*	0904
*Parkia biglobsa* (Jacq.) Benth.	Néré	*fruit, stem bark*	*DC, M*	*well-being, UTI, malaria-symptoms*	0285
*Stylosanthes erecta* P.Beauv	Sekoufali	*leaves*	*DC*	*tiredness, malaria-symptoms*	1401
*Trichilia emetica* Vahl	Sulafinzan				0561
*Vepris heterophylla* (Engl.) Letouzey	Kita kinkeliba	*bundles (mainly leaves)*	*DC*	*malaria-symptoms, constipation, edema*	2444
*Ximenia americana* L.	Ntonké/Hongbé	*fruit, bundles (mainly leaves)*	*DC, vapor*	*well-being, to increase appetite*	0764

DC=decoction, M= maceration
